# Supercapacitors with cotton shell-derived activated carbons and porous polymer electrolyte films†

**DOI:** 10.1039/d5ra00696a

**Published:** 2025-04-01

**Authors:** Saurabh Singh, Yulin Zhang, S. A. Hashmi, Fuqian Yang

**Affiliations:** a Materials Program, Department of Chemical and Materials Engineering, University of Kentucky Lexington KY 40506 USA ssi303@uky.edu; b Chongqing Institute of Green and Intelligent Technology, Chinese Academy of Sciences Chongqing 400714 China; c Department of Physics and Astrophysics, University of Delhi Delhi 110007 India

## Abstract

Some of the well-known challenges in the field of supercapacitors (SCs), or more specifically, electrical double-layer capacitors (EDLCs), such as low energy density and high cost, have proven to be major barriers to their widespread market success despite having some excellent electrochemical merits such as high-power density and good cyclic stability. In this work, efforts have been made to overcome these gaps and eventually enhance the performance of EDLCs *via* a cost-effective and eco-friendly approach. To fabricate these EDLCs, a bio-waste, namely, cotton-shell-derived activated carbons (ZnACs) (activated with ZnCl_2_), was used in a mass ratio of 1 : 2 for cotton shell to ZnCl_2_. This resulted in a large BET surface area of 2031 m^2^ g^−1^ and a hierarchical porous structure, which contributed to faster diffusion of electrolyte ions. These two features ultimately resulted in a high specific capacitance of 247.82 F g^−1^ at a current density of 0.52 A g^−1^ of the cell with a porous polymer electrolyte (PPE) film made from polycaprolactone and poly (vinylidene fluoride-*co*-hexafluoropropylene), which offered the advantages of a wider potential window (∼7.22 V *vs.* Ag) and high conductivity (1.51 mS cm^−1^). A comparison was then made with another cell using commercial activated carbon powder and the same PPE film. The ZnAC-based EDLC cells showed better performances, such as a high energy density (∼22.58 W h kg^−1^) and high Coulombic efficiency (∼83.6%) without compromising the effective power density (∼0.42 kW kg^−1^). EDLC cells exhibited only ∼3% capacitance fading at the end of 10 000 charge–discharge cycles. Thus, the incorporation of cotton shell-biowaste resulted in a two-way advantage of reducing environmental pollution caused by their large-scale burning practices and delivering substantial electrochemical performance, ultimately opening new avenues in the field of green energy technology.

## Abstract 2

Pictorial summary of the characterization of SC cells based on cotton shell-derived activated carbons (ZnACs) and porous polymer electrolytes (PPEs).

## Graphical abstract

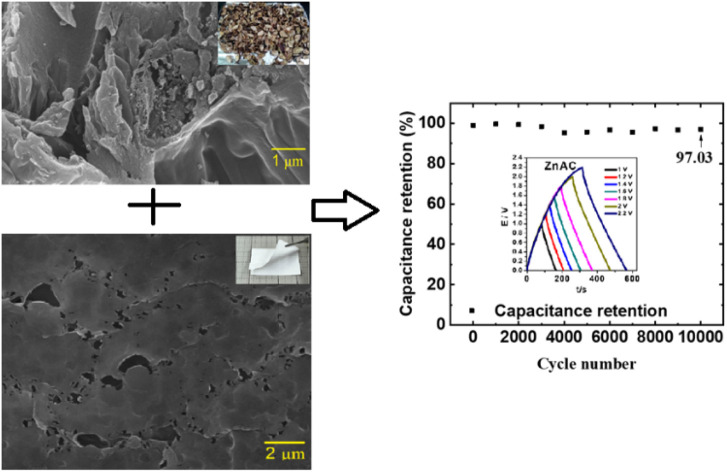

## Introduction

Supercapacitors (SCs) or ultracapacitors have drawn great attention globally in the broad field of energy storage devices owing to their superior characteristics such as high charge–discharge rate, high power density and long cycling performance. Supercapacitors are classified into three categories: electrical double-layer capacitors (EDLCs), pseudocapacitors and hybrid capacitors, with each of these having their own advantages and disadvantages.^1,2^ Among these, a substantial amount of attention has been given on EDLCs in the past few years owing to some of their lucrative characteristics such as high power density and good cyclic stability, which make them superior to pseudocapacitors, even though the latter fill up many loopholes of the former, such as relatively low energy density.^1–6^ Recently, some serious efforts have been invested towards the performance enhancement of EDLCs by mitigating these loopholes through a variety of approaches, such as using different types of electrode materials and different types of electrolytes having wider potential windows.^7–18^

In recent years, researchers have focused on multiple parameters to increase the energy density, specific capacitance, and efficiency of EDLCs without compromising the long-term stability and power density of the cells. First, the focus was on the appropriate selection of electrode materials for SC cells that can provide high specific surface area, good electronic conductivity at low cost while being eco-friendly. Based on these requirements, carbon-based materials emerged as one of the most promising electrode materials for SCs.^1,2,7–10,19^ For commercial purposes, instead of using precursors such as coal and petroleum coke, carbon-based materials are usually derived from natural biomasses owing to their merits such as low production cost, renewability and environment-friendly nature.^20–24^ Selecting an appropriate biomass from a variety of available natural biomasses is challenging as different biomasses contain different lignocellulosic contents, which affects the porosity of materials owing to varying ratios of micro- and meso-pores present in their interior. A variety of biomasses have been tested and their superior results have been reported in the past by different researchers working actively in this field, *e.g.*, Chen, Kang, Wumaier, Dou, Gao, Han, Xu, Liu and Zhang^7^ utilized cotton stalks as precursors and reported a specific capacitance of ∼114 Fg^−1^. Kim, Lee, Kim and Yang^8^ reported a specific capacitance of 5–60 Fg^−1^ based on bamboo-derived precursor. Hor and Hashmi^10^ reported a specific capacitance of 126–146 Fg^−1^ obtained through pollen-cone-based EDLCs. Cao, Liao, Zhang and Chen^25^ reported a specific capacitance of ∼297.5 Fg^−1^ obtained *via* a peasecod-based precursor. Some other precursors include hemp,^17^ potato,^18^ coconut shells,^9^ and coffee shells.^26^ Overall, these studies showed the potential of natural biomasses for their application in SCs/EDLCs.

Significant attention has been paid to choosing an appropriate electrolyte having a wider working potential window. In comparison to other electrolytes, solid polymer electrolytes (SPEs) have been preferred due to their superior characteristics such as wider potential window, low tendency of dendrite formation, light weight and good mechanical properties including flexibility.^27–29^ Polymer electrolytes are classified into three categories: gel polymer electrolytes (GPEs), dry solid polymer electrolytes (DSPEs or SPEs) and porous polymer electrolytes (PPEs).^30–33^ PPEs (or activated porous polymer (APP) films, soaked in a liquid electrolyte) offer both the properties of providing ion-transport channels and good interfacial wetting between electrodes and electrolytes. Some of the other merits of porous polymer (PP) films over commercial separators have been reported. For instance, Zhu, Yang, Fu and Wu^34^ reported superior characteristics of PVDF-HFP-based porous membranes, such as higher tensile strength, higher ionic conductivity (∼4 times), better transfer of electrolyte ions, and better electrolyte retention, to commercial separators. Similar observations have been reported by Yang, Chang, Li, Wang and Wu,^35^ who reported a higher ionic conductivity of 0.60 mS cm^−1^ and a higher ion transfer number of 0.30 for sodium ions in gel polymer electrolytes for the PVDF-HFP-based porous membrane than commercial separators. A significant amount of work showing superiority of porous polymer membranes over commercial separators in terms of better conductivity, better porosity, better electrolyte uptake, *etc.*, has been reported in the literature.^34–36^

For our present work, we chose the biomass of cotton shells, with the botanical name *Gossypium arboreum* (also known as tree cotton) and having a structural composition of ∼38% cellulose, ∼12% lignin and ∼23% hemicellulose,^37^ which is technologically attractive due to three main reasons: (a) easy and large-scale availability (especially around tropical and subtropical regions of world like India and Pakistan),^38^ (b) decent amounts of carbon content (∼45% by weight) present inside cotton shells making them a potential precursor to derive activated carbons (ACs),^38^ and (c) prevention of environmental pollution caused by large-scale burning of these shells. We focused on optimizing the mass ratio of precursor (biomass) to the activating agent, which ultimately controls the fractions of micro and mesoporous interiors, present inside AC powder to obtain higher specific capacitance. The phase separation method has been used to prepare PP films with PCL and PVDF-HFP, which are then activated with an organic liquid electrolyte.^11,39–42^

Overall, in this work, we noted the superiority of cotton-shell-derived activated carbon (ZnAC2)-based supercapacitor cells (SCs) over commercial activated carbon (CmAcs)-derived SCs with a porous polymer electrolyte (PPE) in terms of better electrochemical performance, namely, a higher specific capacitance of 247.82 F g^−1^ (at a current density of 0.52 A g^−1^), a higher energy density of ∼22.58 W h kg^−1^, and a higher Coulombic efficiency of ∼83.6% without compromising the effective power density (∼0.42 kW kg^−1^). The EDLC cells have only ∼3% capacitance fading at the end of 10 000 charge–discharge cycles indicating their long-term stability.

## Experimental details

### Materials

Zinc chloride (purity ∼95%, Merck), acetylene black (AB), polymers of PVDF-HFP (M.W. ∼400 000) and PCL (M.W. ∼45 000), sodium perchlorate (NaClO_4_, purity ∼98%), and organic solvents of propylene carbonate (PC, purity ∼99.7%) and ethylene carbonate (EC, purity ∼98%) were procured from Sigma-Aldrich. *N*-Methyl-2-pyrrolidone (NMP, purity >99.9%) was purchased from Spectrochem. No further purification was done before using these chemicals. Raw cotton shells were collected from the northern parts of India.

### Methods

The raw cotton shells were first cleaned to remove extra cotton and dirt from their surfaces. They were then washed thoroughly with deionized (DI) water and dried in an oven at ∼80 °C, following which they were ground into fine powders. Mixtures consisting of cotton shell powder and ZnCl_2_ in a mass ratio from 1/1 to 1/4 were mixed with DI water, respectively. Continuous stirring was done for nearly 7–8 hours to obtain a homogeneous mixture, following which the mixtures were dried in a vacuum oven at ∼110 °C. Once the mixtures were dried completely, they were put into alumina boats after which these boats were placed in a tube furnace. Heating the mixtures was performed by increasing the temperature from room temperature to ∼800 °C at a rate of 5 °C min^−1^ under continuous flow of nitrogen gas (N_2_). After reaching ∼800 °C by heating for 20–30 min, the N_2_ gas was replaced with CO_2_ gas for physical activation of the mixtures at ∼800 °C for 2–3 hours. Note that chemical activation of the cotton shell powders with ZnCl_2_ took place first during heating and etched the formed carbon powders to obtain a large specific surface area. The physical activation widened the pores of carbon powders. Finally, the carbon powders (activated carbons (ACs)) were collected from the tube furnace after the temperature of the furnace dropped to room temperature. A 5% HCl solution (V/V) was used for washing the carbon powders followed by continuous washing with DI water to remove impurities such as zinc and chloride ions till the waste solution reached neutral state. After washing, the AC powders were dried in a vacuum oven for ∼15 h at ∼110 °C. We termed the prepared ACs with ZnCl_2_ as “ZnACs”.

PP films were prepared from PCL and PVDF-HFP in a ratio of 9/1 (w/w) using a phase separation method. Briefly, a thick polymer solution of PCL/PVDF-HFP and NMP in a ratio of 1/6 (w/w) was prepared and casted as a thin film onto a glass plate of 10 × 10 cm^2^ dimensions using a micro-meter adjustable film applicator (MTI corporation). The glass plate with the film was immersed in water (which acts as a non-solvent here) for 3–4 minutes. Polymer-rich and -poor phases were formed throughout the film due to precipitation caused by the exchange of solvent with non-solvent. After immersion, the films were left in an open environment for ∼15 min to remove the excess water present inside. This was followed by vacuum drying at ∼55 °C, and the dried films were kept in an inert atmosphere for further use. The PP films were activated using an organic liquid electrolyte, 1 M NaClO_4_, in EC : PC (1 : 1 V/V). The obtained activated PP (APP) films were used in the fabrication of SC cells.

### Characterization techniques

The characterization of the ZnACs was performed. The specific surface area measurements and porosity analyses were carried out by N_2_-adsorption–desorption (at ∼77 K) using a surface area and pore size analyser (Gemini-V, Micromeritics, Norcross, USA). A scanning electron microscope (SEM) and a field-emission scanning electron microscope (FESEM) (Zeiss Gemini SEM 500, Carl Zeiss, Germany) were used to study the morphology of ZnACs. XRD patterns were obtained using an X-ray diffractometer (D8, Brucker, USA) with CuK_α_ radiation of wavelength *λ* ∼1.5406 Å at a scan rate of 2° per minute. The Raman spectra of carbon samples were recorded using a spectrometer (inVia Reflex, Renishaw, UK) coupled with a laser of 532 nm wavelength.

Morphological studies were conducted on the prepared PP films using a field emission scanning electron microscope at a potential difference of 20 kV. The ionic conductivity and electrochemical stability window (ESW) of the APP films were measured using a CHI660E electrochemical workstation (CH instruments). The ESW of the APP films was evaluated using a linear sweep voltammetry (LSV) technique based on a configuration in which the APP film was sandwiched between a stainless steel (SS) foil (as working electrode) and a silver (Ag) foil (as reference electrode). The ionic conductivity of the APP film was evaluated by electrochemical impedance spectroscopy (EIS) in the frequency range from 100 kHz to 10 mHz with an AC voltage amplitude at 10 mV.

### Electrode fabrication

AC-electrodes were prepared from ZnACs and commercially available ACs (CmACs). For the preparation of AC electrodes, the optimized ZnACs (or CmACs) were mixed with a conductive additive (acetylene black) and a polymer binder (PVDF-HFP) in a weight ratio of 80/10/10. Acetone was added to the mixtures to form slurries, after which they were coated onto graphite sheets (∼250 μm thick, Nickunj Eximp Enterprises, India). The finally prepared electrodes of 15 mm diameter were dried in a vacuum oven at ∼100 °C for 13–14 h before use in SC cells.

### Supercapacitor cell fabrication

SC cells were constructed using APP films sandwiched between two symmetrical AC-electrodes. The construction was done in a two-electrode split test cell (MTI Corporation). Two different EDLC cells were constructed, using ZnAC and CmAC electrodes and APP films, which are presented below:Cell-1: (ZnAC)|APP|(ZnAC)Cell-2: (CmAC)|APP|(CmAC)

The loading mass of ACs was 1.92 mg per electrode. The SC cells were characterized using an electrochemical workstation (CH1660E, CH instrument) for electrochemical impedance in the frequency range of 100 kHz to 10 mHz and the CV tests, whereas GCD tests were conducted using a charge discharge unit (BT-2000, Arbin Instruments).

## Results and discussion

### Characterization of PP and APP films

**Fig. 1:**
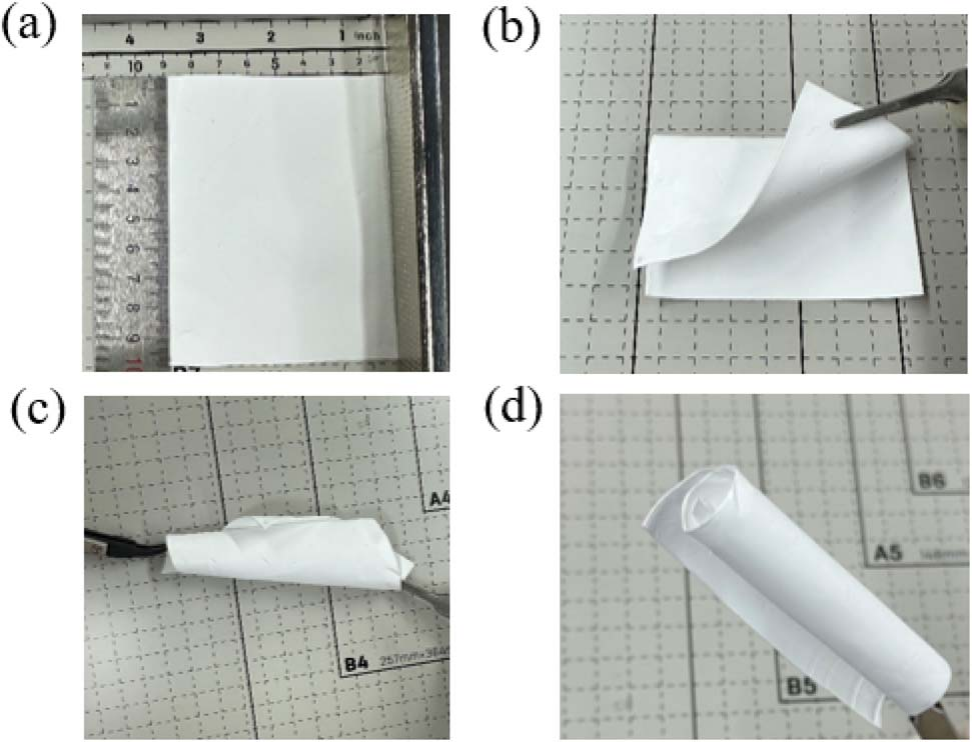
(a–d) Optical images of the prepared PP films at different deformation states.

Fig. 1a shows the optical images of a prepared PP film of thickness ∼0.25 mm in the form of a free-standing film. Fig. 1b–d show the prepared PP films at different deformation states, namely, twisting, folding and multiple folding, for the demonstration of their flexibility. It is observed that the PP films are not too fragile and can easily be folded multiple times without breaking, indicating their mechanical flexibility. Fig. S1e and f† show the FESEM image of a PP film with a dense porous structure. Pores are present in arbitrary shape, and the pore ranges from 2 to 10 μm.

The n-butanol adsorption test was used to determine the porosity of the prepared PP films.^43^ The butanol uptake of the prepared PP films was recorded with respect to time, and the respective porosity was calculated using the following equation:^43^1
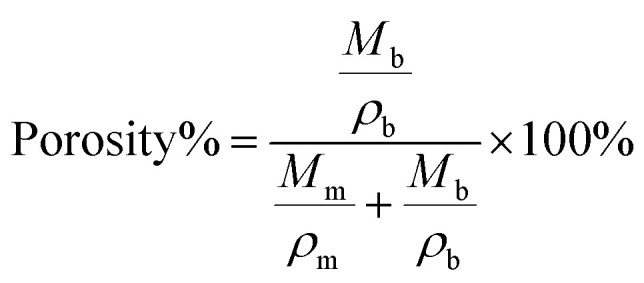
where *M*_m_, *M*_b_, *ρ*_m_ and *ρ*_b_ correspond to the masses and densities of dry membranes and absorbed *n*-butanol in the prepared PP films, respectively.

**Fig. 2:**
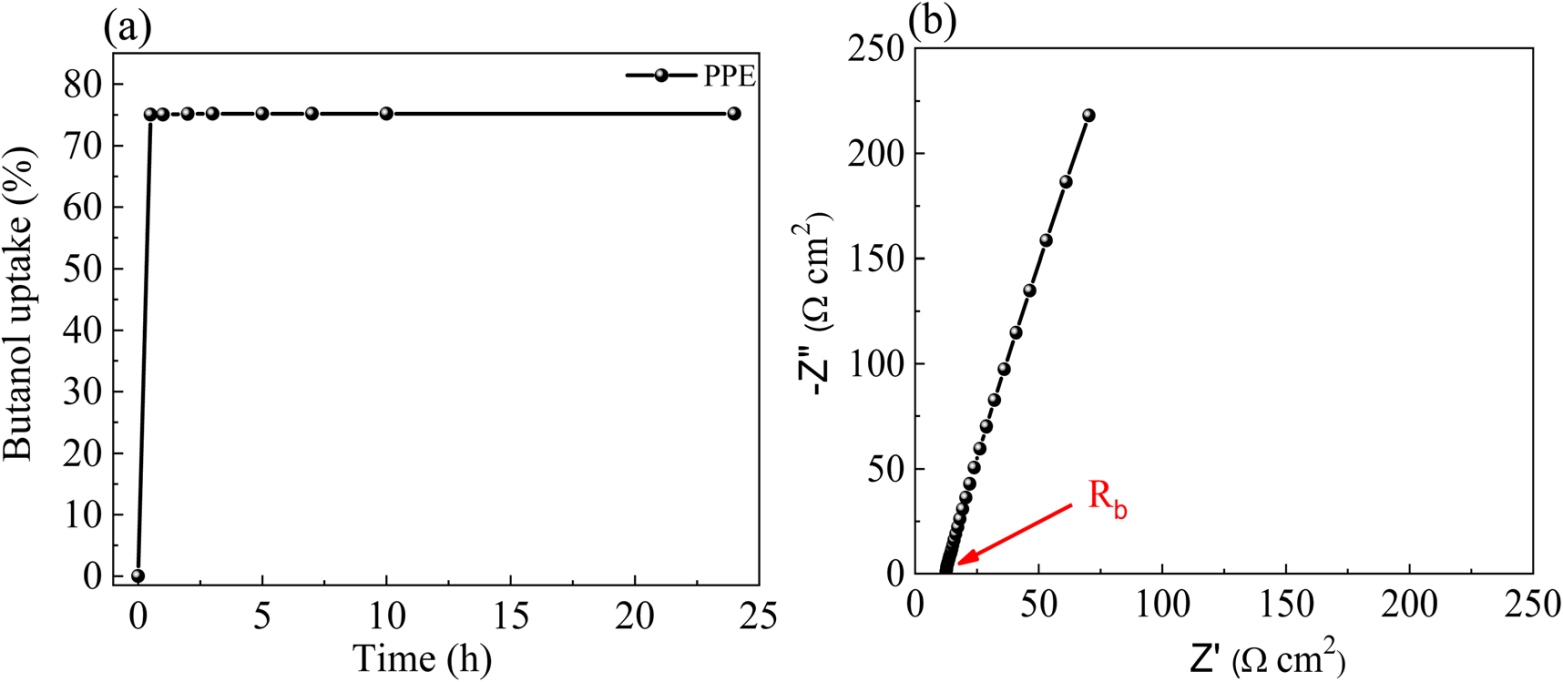
(a) Temporal evolution of butanol uptake (%) for a PP film and (b) EIS plot of an APP film.

Fig. 2a shows the temporal evolution of butanol uptake (%) in a PP film. There is a sudden uptake of *n*-butanol in the first ∼30 min, following which the PP film got saturated. The porosity was found to be ∼75% from eqn (1), which is sufficient to accommodate liquid electrolytes.

Fig. 2b shows the EIS plot of an APP film, from which the bulk resistance (*R*_b_) of the APP film was calculated to be 12.46 Ω. The ionic conductivity of APP can be evaluated using the following formula:2
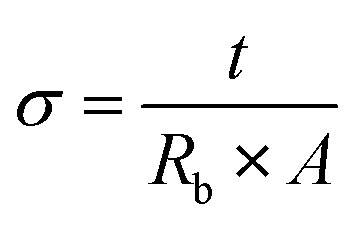
with *t* and *A* as the thickness and area of the film, respectively. Using *t* = 0.25 mm and *A* = 1.327 cm^2^, we obtained 1.51 mS cm^−1^ for the ionic conductivity of the APP film. Note that the ionic conductivity of the APP film is mainly due to the Na^+^ and ClO_4_^−^ ions of the organic electrolyte.

**Fig. 3:**
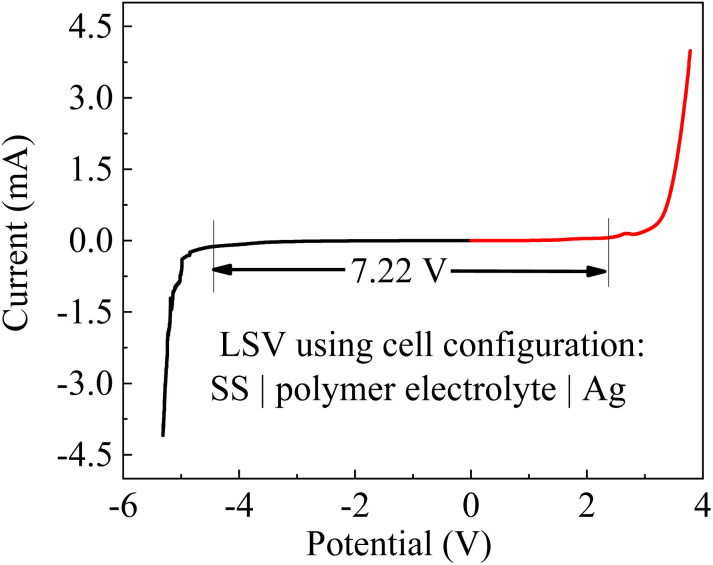
*I*–*V* curve for the cell configuration of SS|APP film|Ag at a scan rate of 10 mV s^−1^.

Fig. 3 shows the *I*–*V* curve of the sandwich structure of SS|APP film|Ag, indicating that the APP film is electrochemically stable up to ∼7.22 V *versus* Ag at room temperature. The cathodic stability is up to ∼−4.4 V, and the anodic stability is up to ∼2.3 V. The asymmetry in the anodic and cathodic potential window is due to the difference in the oxidation and reduction potential ranges. The wide potential window of the APP films makes them appropriate for application in energy storage.

### Characterization of ACs

**Fig. 4:**
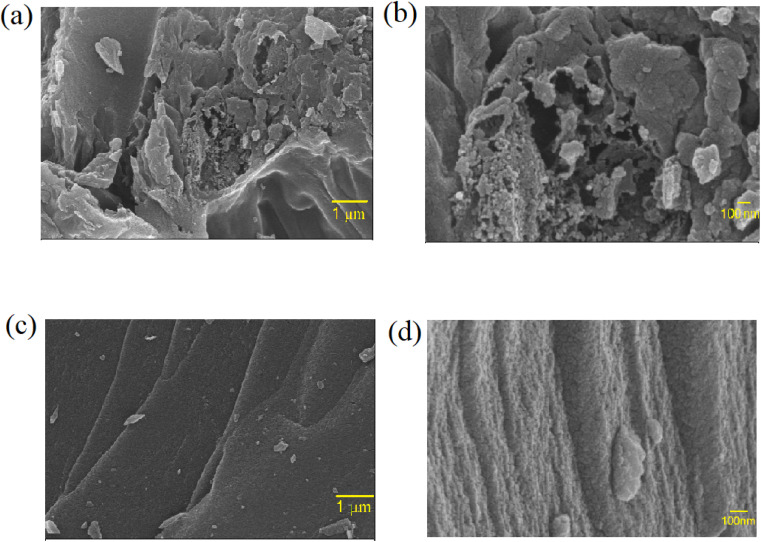
FESEM images of ZnACs (a and b) and CmACs (c and d).

The carbon content in ZnACs was found out to be ∼98%, which is much higher than the amount found in CmACs (∼72%), as characterized through Energy-Dispersive X-ray Analysis (EDAX), indicating the preferability of ZnACs over CmACs for SC electrodes. Moreover, the FESEM images of ZnACs and CmACs are shown in Fig. 4a–d, respectively. There are pores with a size less than 1 μm in the ZnACs, whereas no pores are visible in CmACs. The surface of the ZnACs is rougher and has more pores than the CmACs. Such a result suggests that electrolyte ions can migrate through the pores of ZnACs easier, leading to an increase of electrolyte ions stored in ZnACs and the fast migration of electrolyte ions during electrochemical cycling.

**Fig. 5:**
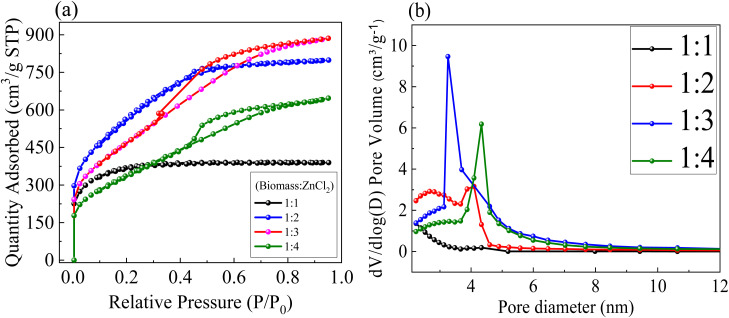
(a) N_2_-adsorption–desorption isotherms and (b) pore-size distribution for ZnACs.

Fig. 5a shows the N_2_-adsorption–desorption isotherms of ZnACs prepared with four different mass ratios of cotton shell powders to ZnCl_2_ (1 : 1, 1 : 2, 1 : 3 and 1 : 4). There is a rapid increase in the adsorption for the relative pressure *p*/*p*_0_ < 0.005, which is followed by a gradual increase in the adsorption within the relative pressure range of 0.005 < *p*/*p*_0_ < 0.5. Such a result points toward the presence of a substantial amount of micro- and meso-porous pores in the ZnACs. According to the IUPAC classification, the ZnACs (1 : 1) exhibits Type-I isotherm, indicating negligible porosity in the ZnACs because of the low amount of ZnCl_2_ involved in the activation. The other ZnACs (1 : 3 and 1 : 4) exhibit Type-IV isotherms, indicating the presence of a large amount of meso-pores in them. The ZnACs (1 : 2) exhibit a combination of Type I and Type IV isotherms,^44,45^ indicating the presence of both micro- and meso-pores.

From the isotherms, we calculated the BET specific surface areas (SSA) and average pore sizes of the ZnACs, as listed in Table 1. It is evident that ZnACs (1 : 2) have a maximum BET SSA in comparison to other ZnACs. Using a smaller or greater amount of activating agent than the desired amount has a negative effect on the BET SSA of the cotton shell-based ZnACs. As seen in Fig. 5a and b, if the ZnCl_2_ amount if lower (1 : 1), it resulted in insufficient activation, leading to less adsorption sites and formation of less pores, which overall decreases the effective surface area. If ZnCl_2_ is excessive (1 : 3 and 1 : 4), it leads to over-activation, resulting in possible pore collapse along with pore blockage, which ultimately reduces the effective surface area. Only when the amount of ZnCl_2_ is optimal, it leads to an appropriate balance of micro- and meso-pores, overall leading to a higher effective surface area proving the optimal accessibility for electrolyte ions. Similar observations indicating the optimal behaviour of the 1 : 2 sample, as compared to other ratios, have been reported many times in past literature reports also.^10,46^ For instance, Hor and Hashmi^10^ tested different ratios of pollen-cone char powder to ZnCl_2_, and after filtering through BET analysis, the 1 : 2 sample was found to have a maximum surface area and appropriate porosity, making them suitable electrode materials, and hence, all other electrochemical characterizations were performed on this 1 : 2 sample only which was further utilized for making SC electrodes. Using the BJH (Barrett–Joyner–Halenda) method, we calculated the average pore sizes of the prepared ZnACs. Fig. 5b shows the distribution of pore sizes of the ZnACs. The average pore size of ZnACs (1 : 2) is ∼2.4 nm (Table 1), which is sufficient for the migration of electrolyte ions through pores in ZnACs (1 : 2). Hence, due to the appropriate balance between micro- and meso-pores in the 1 : 2 sample, leading to not only a higher specific surface area but also optimum accessibility routes for electrolyte ions migration, we constructed SCs with this mass ratio of 1/2 in the rest of the studies and all other characterizations that involved the incorporation of this mass ratio.

**Table 1:** Surface area and average pore size of ZnACs

Material	Mass ratio of AC to ZnCl_2_	S_BET_ (m^2^ g^−1^)	Average pore size (nm)
ZnAC1	1 : 1	1207	1.9
ZnAC2	1 : 2	2031	2.4
ZnAC3	1 : 3	1694	3.2
ZnAC4	1 : 4	1215	3.3

**Fig. 6:**
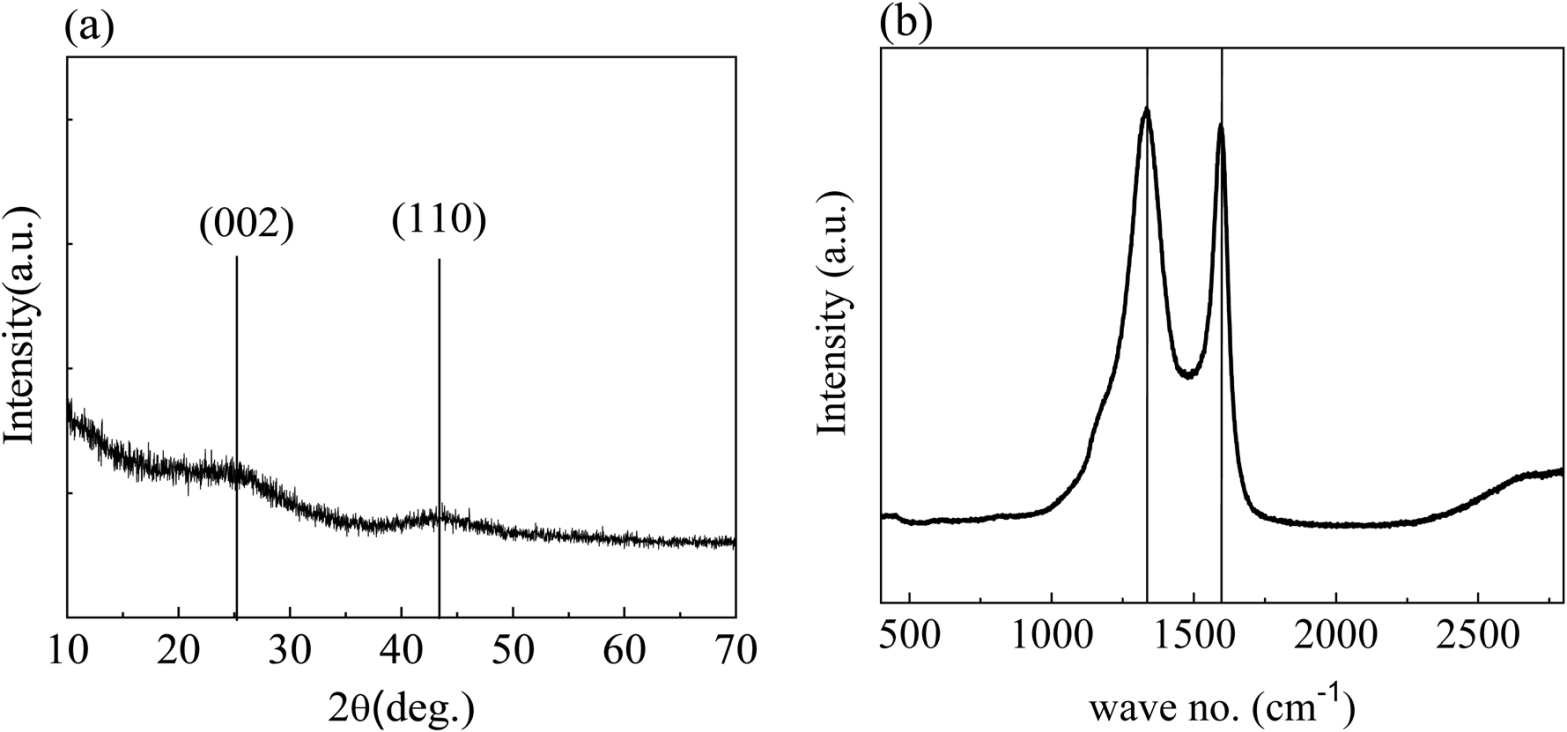
(a) XRD pattern and (b) Raman spectrum of ZnACs (1 : 2).

Fig. 6a depicts the XRD pattern of ZnACs (1 : 2). There are two prominent broad peaks centred at 2*θ* = ∼24.5° and ∼43.0°, which correspond to the (002) and (100) planes of carbon, respectively. The broad peaks suggest the amorphous nature of ZnACs (1 : 2). The peak centred at 24.5° can be used to calculate the pseudo-graphitic interlayer spacing (*d*_002_) and average crystallite height by the Debye–Scherrer formula. Note that this formula can also be used to calculate the average crystallite width from the peak centred around 43.0°.^47^ Using the XRD pattern in Fig. 6a, we obtained ∼3.6 Å for *d*_002_, which is larger than 3.35 Å for graphite.^48^ This result indicates that the prepared ZnACs (1 : 2) have turbostratic (fully disordered) characteristics.^49^ The broad and weak peak centred around 43.0° indicates that there is less development of intra-graphitic layer in the ZnACs (1 : 2). Moreover, XRD studies are expected to show a similar pattern for all other three ratios, as indicated in previous literature reports.^50,51^ For instance, Rajbhandari, Shrestha, Pokharel and Pradhananga^50^ observed that the XRD pattern is nearly the same irrespective of different impregnation ratios of ZnCl_2_ and only their amorphous behaviour was being through these XRD studies. Hence, the characterization was performed only on 1 : 2 samples, and the amorphous highly disordered nature depicted through their XRD analysis supported presence of significant porosity inside them, making them suitable precursors for SC electrodes.

Fig. 6b shows the Raman spectrum of ZnACs (1 : 2). There are two dominant peaks around 1335 cm^−1^ and 1592 cm^−1^, which correspond to the D-band and G-band, respectively. The intensity ratio of D-band to G-band (*I*_D_/*I*_G_) tells us the degree of ordered carbon present inside the sample with *I*_D_/*I*_G_ > 1 indicating the presence of highly disordered and defect-rich carbon and *I*_D_/*I*_G_ < 1 indicating a more graphitic behaviour. For ZnAC (1 : 2), this intensity ratio (*I*_D_/*I*_G_) turned out to be ∼1.04 due to the higher intensity of D-band (as shown in Fig. 6b), revealing a higher degree of disorderedness,^52^ making them suitable electrode materials for supercapacitor applications. Moreover, for all other three ratios, a similar pattern of Raman spectra is expected based on past literature reports.^50,51^ For instance, Rajbhandari, Shrestha, Pokharel and Pradhananga^50^ noted that the intensity ratio of G to D bands in Raman spectroscopy are independent irrespective of the impregnation ratio of ZnCl_2_, carbonization temperature and precursor source. Hence, our studies are conducted only on the 1 : 2 sample to depict the disordered behaviour of this type of activated carbon.

It is also worth noting that neither XRD nor Raman spectra depicted the presence of Zn moieties due to either removal of all possible Zn moieties entirely during multiple washing of the sample with 5% HCl solution or highly disordered nature of any residual Zn moieties, which cannot be detected due to limitations of instrument. This is a common phenomenon being reported multiple times in the literature.^50,51,53^

### Electrochemical performance of SC cells with the APP film

**Fig. 7:**
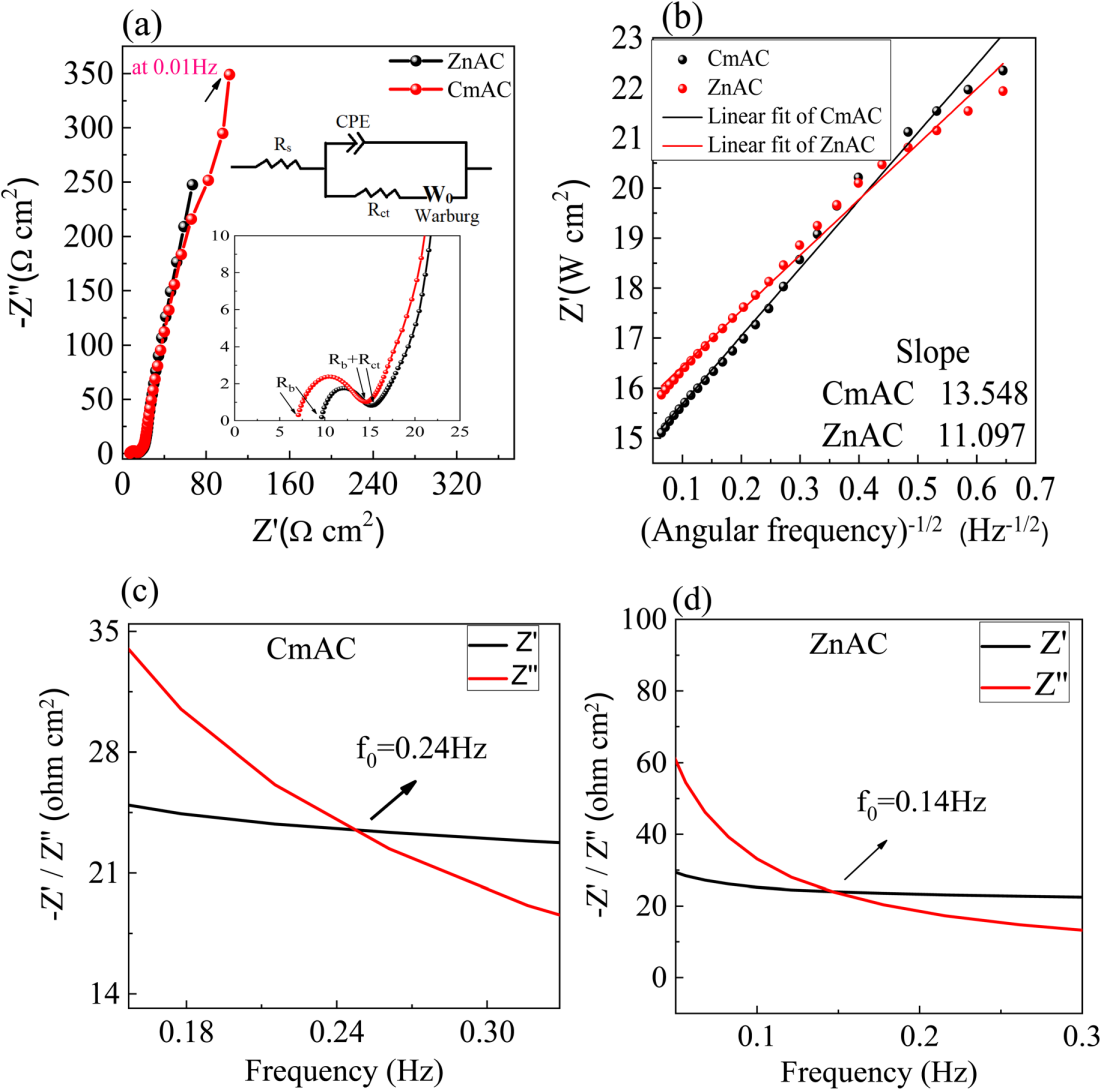
(a and b) Nyquist plots with the inset depicting the equivalent circuit used to fit curves and (c and d) Bode plots of the prepared SC cells.

Fig. 7a and b display the Nyquist plots of the SC cells with ZnACs as the electrode material. For comparison, the Nyquist plots of the SC cells with CmACs as the electrode material are also included in the corresponding plots. There exists a sharp increase in the imaginary impedance in the low-frequency range for the respective SC cells made from ZnACs and CmACs, indicating the capacitive characteristics of the SC cells.

In the medium-frequency region, the segment of the Warburg type line (45° region) is related to ionic diffusion in the associated SC cells.^54^ The SC cells with ZnACs exhibit a shorter segment than that of the SC cells with CmACs, revealing that the SC cells with ZnACs have smaller pathways for ionic diffusion attributed to the micro- and mesopores in ZnACs. In the high-frequency region, the semicircular variation of the imaginary impedance with the real impedance (the inset in Fig. 7a) indicates the RC characteristics of the SC cells with the series resistance consisting of intrinsic resistance of the electrode material, electrolyte resistance, and the contact resistances between the electrode and the electrolyte and between the electrode and the current collector. Fig. 7b shows the lumped circuit used to fit the Nyquist plots shown in Fig. 7a. The fitting curves are included in Fig. 7a. It is evident that the lumped circuit describes well the electrochemical impedance of the SCs. The variation in the real impedance with the square root of angular frequency is shown in Fig. 7b as well as the results derived from the fitting results of the lumped circuit, demonstrating the applicability of the lumped circuit. Fig. 7c and d show the Bode plots of the respective SC cells made from CmACs and ZnACs. The critical frequency, *f*_0_, at which both real and imaginary parts of impedance coincide, was obtained to be 0.24 and 0.14 Hz for the SC cells made from CmACs and ZnACs, respectively.

Table 2 summaries the numerical values of *R*_s_ (series resistance), *R*_ct_ (space charge resistance), *σ* (Warburg coefficient), *Z*_w_ (Warburg impedance), *C*_d_ (diffusion capacitance), *f*_0_, and *C*_sp_ (specific capacitance), as calculated from the curve fitting of the Nyquist plots with the lumped circuit. The specific capacitance of SCs as derived from the EIS studies was calculated as follows:^10,11^3
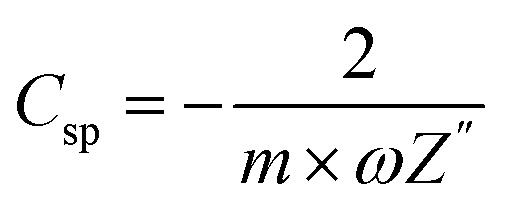
where *ω* is the angular frequency, *Z*′′ is the magnitude of the imaginary impedance at 10 mHz, and *m* (=1.92 mg) is the mass of active material loaded on a single electrode, excluding the mass of the binder (*i.e.* PVDF-HFP) and conductive additive (*i.e.* acetylene black).

**Table 2:** Numerical values of the electrochemical parameters of the SC cells from EIS studies

SCs	*R*_s_ (Ω cm^2^)	*R*_ct_ (Ω cm^2^)	*σ* (Ω cm^2^ Hz^−1/2^)	*Z*_w_ (Ω cm^2^)	*C*_d_ (F)	*f*_o_ (Hz)	*C*_sp_ (F g^−1^)
ZnACs	9.0–10.5	3.5–7.0	11.09	0.94	0.07	0.14	67
CmACs	6.5–7.5	5.5–9.5	13.54	1.14	0.04	0.24	47

According to Table 2, the SC cells with ZnACs depicted better performance with lower numerical values of *R*_ct_ and *Z*_w_ and a higher specific capacitance than CmACs. Such results are due to a higher specific surface area and appropriate pore sizes. The lower value of *R*_ct_ for the SC cells with ZnAC electrodes indicates the favourable behaviour towards faster migration of electrolyte ions into the electrode materials.^55^

**Fig. 8:**
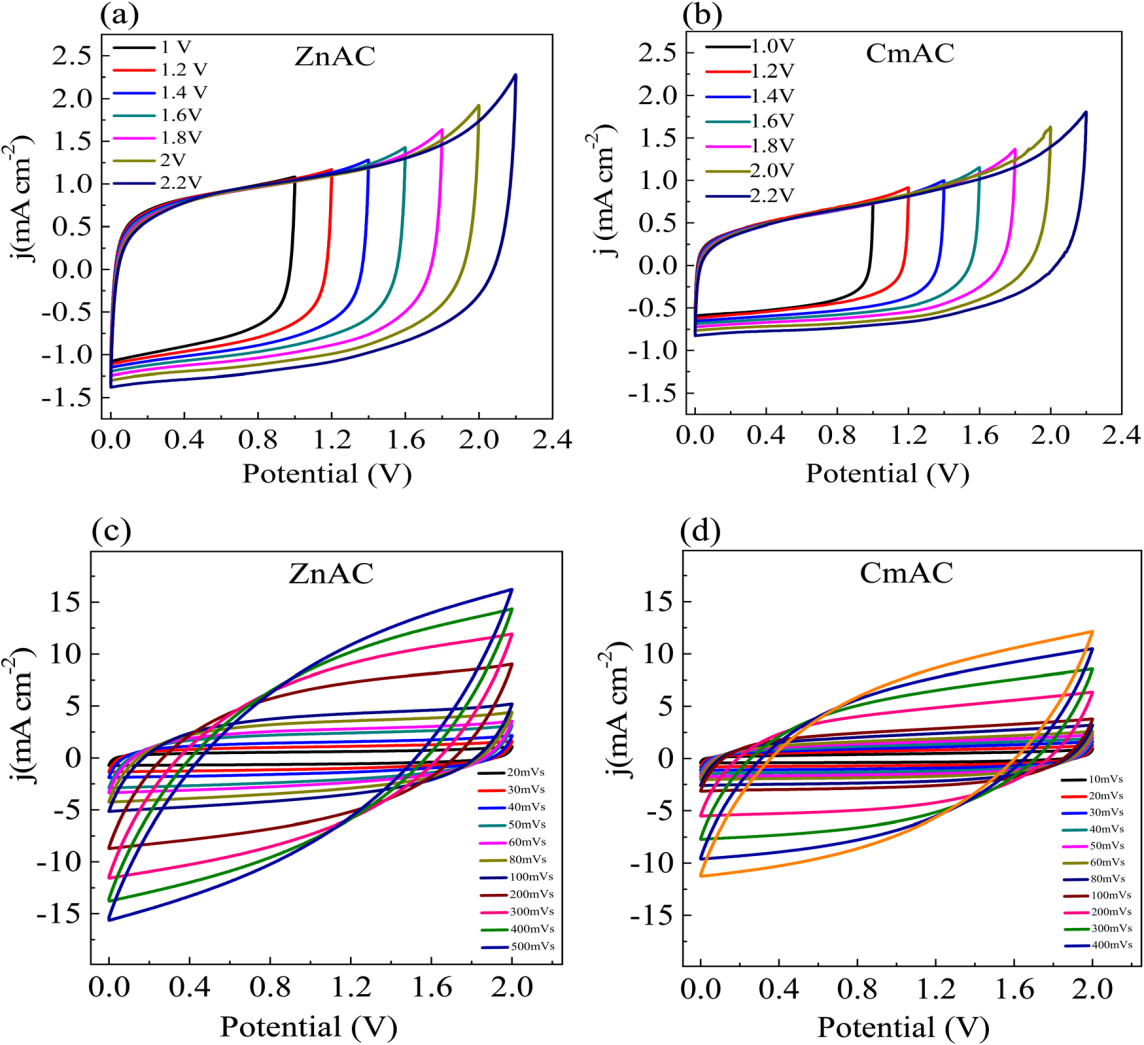
CV curves of the SC cells (a and b) under different potential windows and (c and d) at different sweep rates.

Fig. 8a and b show the CV curves of the SC cells at a sweep rate of 10 mV s^−1^ at room temperature for different voltage ranges. A substantial deviation is observed beyond the potential of 2 V, which concludes that the SC cells have an optimum potential window of 0 to 2 V. Thus, both SC cells were electrochemically cycled in the potential window of 0–2 V in the rest of the studies.

Fig. 8c and d show the CV curves of the SC cells at different sweep rates at room temperature. It is evident that both the SC cells exhibited good CV performance for the sweep rate up to 500 mV s^−1^, which indicates that the SC cells have the high-rate capability, making them useful in practical applications.

From the CV curves, the specific capacitance per electrode was calculated as follows:^56^4
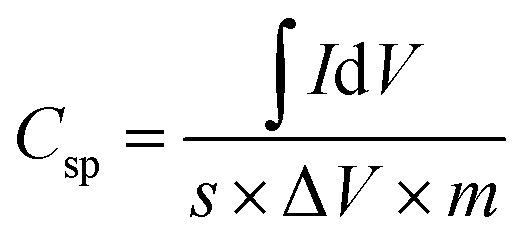
where *I* is the voltammetric current, *V* is the applied potential, Δ*V* is the working potential window, and *s* is the sweep rate. At a sweep rate of 10 mV s^−1^, the specific capacitance for SC cells is ∼108 F g^−1^ with ZnACs as compared to ∼68 F g^−1^ with CmACs indicating the superiority of ZnACs over CmACs for these SC cells. Moreover, this specific capacitance of ∼108 F g^−1^ with ZnACs (1 : 2) is larger than ∼54 F g^−1^ for the SC cells with ACs from *Terminalia catappa* leaf,^57^ ∼68 F g^−1^ for the SC cells with ACs from banana peel,^58^ and comparable to ∼108 F g^−1^ for the SC cells with ACs from coffee leaves.^59^ Hence, cotton shells are a potential precursor for making ACs as electrode materials of supercapacitors.

**Fig. 9:**
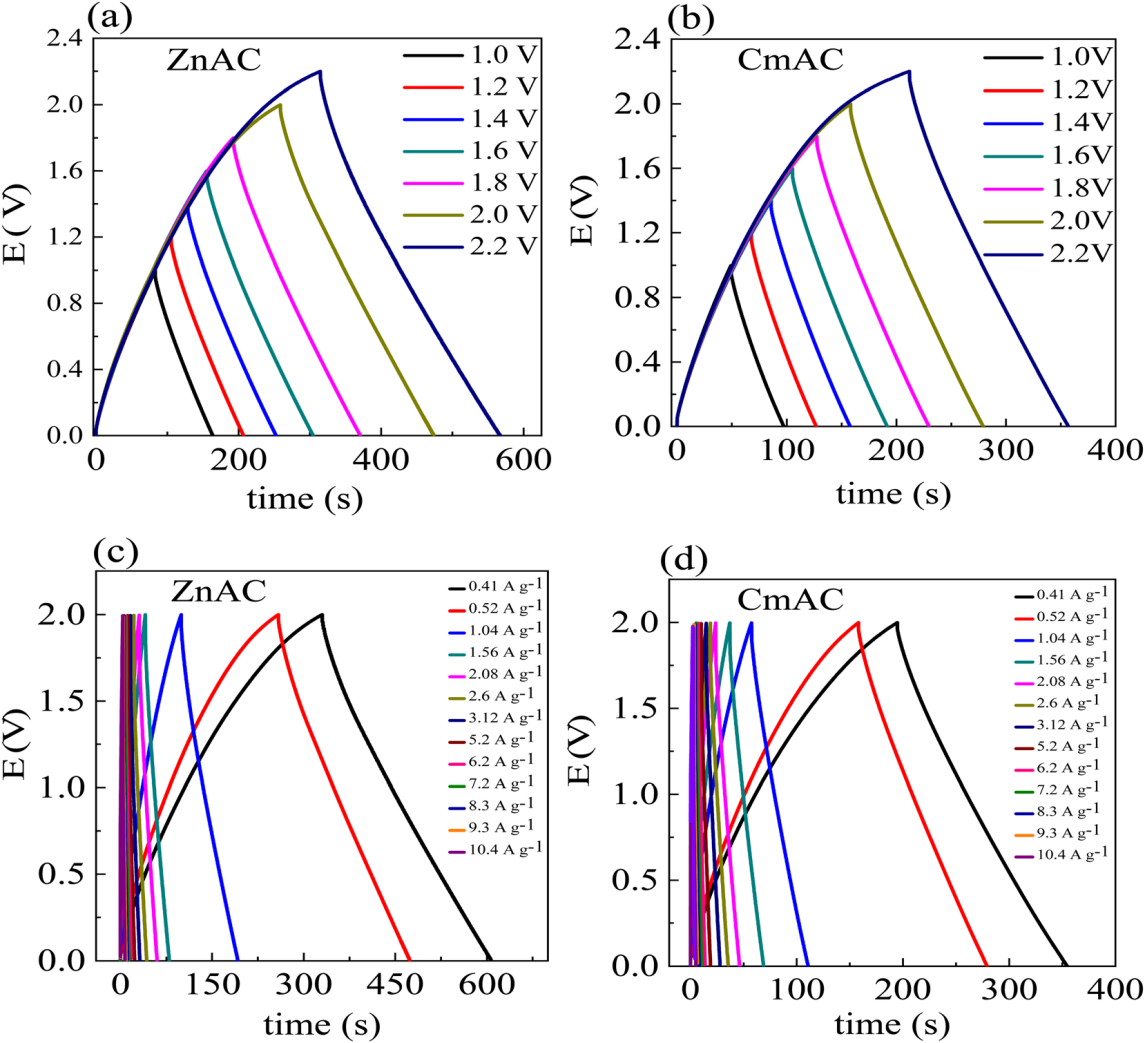
GCD curves of the SC cells (a and b) at a current density of 0.52 A g^−1^ and (c and d) at different densities for the potential window of 0 to 2 V.

Fig. 9a and b show the GCD curves of the SC cells with ZnACs and CmACs at a current density of 0.52 A g^−1^ at room temperature. A considerable deviation from the ideal triangular GCD patterns is noted beyond 2 V, suggesting the potential window in the range of 0 to 2 V. Such a result is in accordance with the one obtained from the CV analysis. Thus, the GCD studies of the SC cells were limited in the potential window of 0 to 2 V.

Fig. 9c and d show the GCD curves of the SC cells with ZnACs and CmACs in the potential window of 0 to 2 V at different current densities. These SC cells are stable up to the high current density of ∼10 A g^−1^. Increasing the current density decreases the charging and discharging times and reduces the specific capacitance of the SCs.

From the GCD curves, we calculated the specific capacitance per electrode using the following equation:^60^5
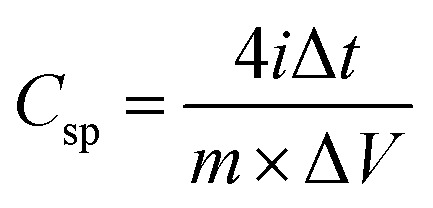
where *i* is the discharge current, Δ*t* is the discharge time interval and Δ*V* is the maximum discharge potential after IR drop. The ESR (equivalent series resistance (https://en.wikipedia.org/wiki/Equivalent_series_resistance)/internal resistance) of the SC cells was calculated using the following equation:6
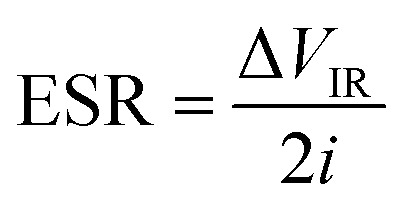
where Δ*V*_IR_ is the IR drop evaluated for initial discharge curve. The specific energy density (*E*_sp_), effective specific power density (*P*_eff_), and Coulombic efficiency (*η*%) of the SC cells were calculated from the GCD curves using the following equation:^60^7
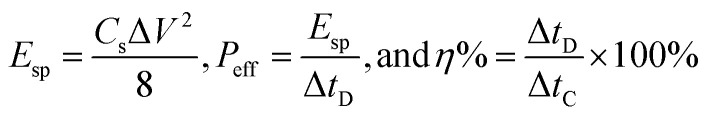
where Δ*t*_C_ and Δ*t*_D_ are the time intervals for constant current charge and discharge, respectively. Table 3 lists the numerical values of the electrochemical parameters calculated from the GCD curves at 0.52 A g^−1^ with the potential window of 0–2 V. It is evident that the SC cells with ZnACs (1 : 2) have higher specific capacitance and energy density than the SC cells with CmACs in accordance with the results from the CV analysis. The SC cells with ZnACs (1 : 2) outperform the SC cells with CmACs in terms of storing and delivering more energy for the same mass, enabling their longer operation without comprising the effective power performance ensuring their prolonged functionality in energy-demanding real-world applications.

**Table 3:** Numerical values of the electrochemical parameters calculated from the GCD curves at 0.52 A g^−1^ with the potential window of 0–2 V

SCs	IR drop (V)	ESR (Ω cm^2^)	*C*_sp_ (F g^−1^)	*E*_sp_ (W h kg^−1^)	*P*_eff_ (kW kg^−1^)	Efficiency (%)
ZnACs	0.38	190	247.82	22.58	0.42	83.66
CmACs	0.4	200	138.69	12	0.40	76.72

**Fig. 10:**
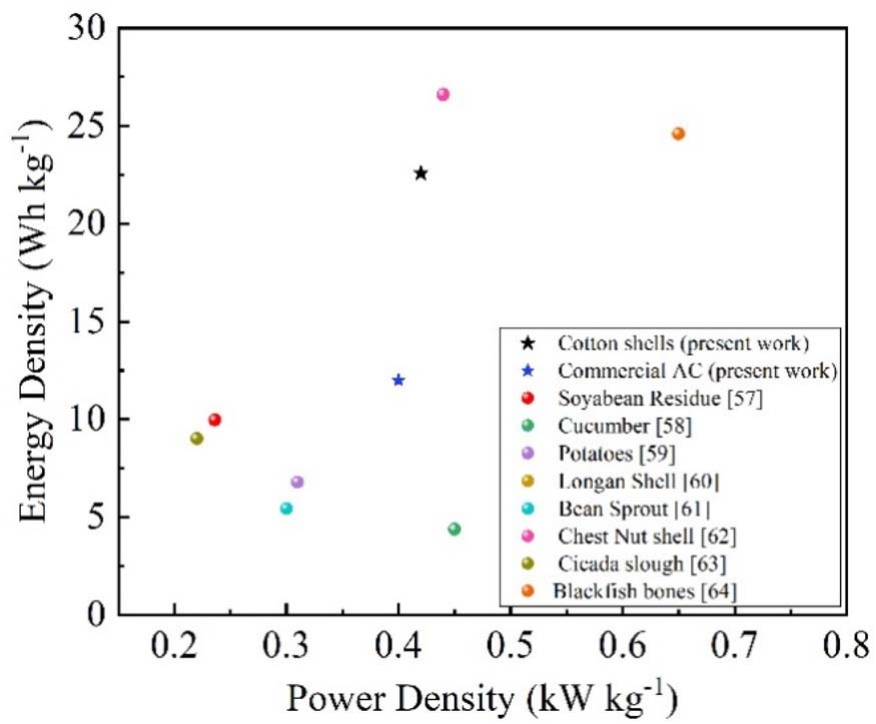
Ragone Plot of supercapacitor cells derived from various precursors at a current density of 0.52 A g^−1^.

Moreover, Fig. 10 shows the Ragone plot for supercapacitor cells utilizing different precursors for activated carbon electrodes. It is pretty evident from the plot that supercapacitor cells based on cotton-shell-derived activated carbons (ZnACs) outperform not only the commercial activated carbons (CmACs), in terms of energy density and power density, but also many other precursors used in past research works showing superiority and importance of this biowaste, unlocking new avenues for its incorporation in different fields of energy storage.^61–68^

For the purpose of comparison, Table 4 lists various electrochemical parameters of SC cells derived *via* different types of ACs and electrolytes, as reported in the literature. It is pretty evident that cotton-shell-derived ACs (ZnACs) dominate over various other precursors reported in the literature in terms of superior electrochemical performance realized in terms of higher specific capacitance and high energy density without compromising effective power density with excellent long-term stability, making them promising precursors for ACs with application as electrode materials of SCs with APP films, and hence, open a possible avenue to improve the performance of SCs.

**Table 4:** Comparison of the electrochemical parameters of SC cells with ACs derived from different precursors and electrolytes

Ref.	Precursor	Electrolyte	SSA (m^2^/g)	*C*_sp_ (F g^−1^)	*E*_sp_ (W h kg^−1^)	*P*_eff_ (kW kg^−1^)	Cyclic stability
69	Native european deciduous trees	1 M H_2_SO_4_	614	24	0.53	0.05	100 (10 000)
10	Pollen cone	ILGPE	1925	126–146	18–21	0.11–0.19	∼90 (10 000)
70	Bamboo shoot	6 M KOH	3300	209	12.6	0.299	95 (10 000)
62	Cucumber	6 M KOH	389	143	4.38	0.45	97 (1000)
71	Rotten carrot	6 M KOH	1253	134.5	29.1	0.14	—
63	Longan shell	6 M KOH	—	210	17.2	1	100 (10 000)
72	Pine nut shell	6 M KOH	956	128	—	—	98 (10 000)
73	Watermelon seeds	NaClO_4_	1920	120	79	22.5	90 (150 000)
74	Coffee seeds powder	3 M KOH	1824	148	12.8	6.64	97 (10 000)
75	Carton box	1 M TEABF_4_/AN	2731	178	45	0.338	96.4 (5000)
Present work	Cotton shells (ZnAC)	PPE	2031	247.82	22.58	0.42	97.03 (10 000)

**Fig. 11:**
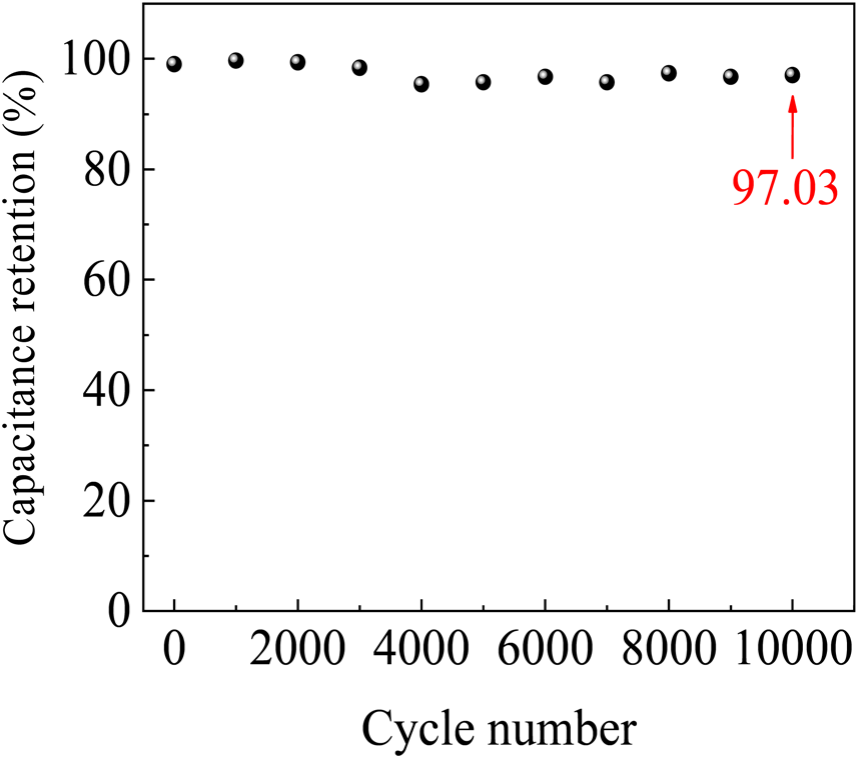
Long-term stability of the SC cells with ZnACs at 1 A g^−1^ in the potential window of 0–2 V.

Fig. 11 shows the long-term stability of the SC cells with ZnACs over 10 000 charge–discharge cycles at 1 A g^−1^ in the potential window of 0–2 V. There is only ∼3% loss of the specific capacitance over 10 000 charge–discharge cycles, suggesting that the SC cells with ZnACs possess long-term stability for the cycling conditions. The capacitance loss is likely due to some irreversible redox reactions at the electrode–electrolyte interface.^76^

Furthermore, there are several reports that tested SC cells with different electrolytes and highlighted their capacitance retention after a specific number of charge–discharge cycles. For instance, Hashmi, Yadav and Singh^14^ reported ∼15% loss of specific capacitance after 10 000 charge–discharge cycles for the SC cells with IL-incorporated PPE. Adan-Mas, Alcaraz, Arévalo-Cid, López-Gómez and Montemor^26^ observed ∼15% loss of specific capacitance after 5000 charge–discharge cycles for the SC cells with coffee-derived ACs. Ma, Guo, Sun, Peng, Yang, Zhou, Zhao and Lei^45^ noted ∼7% loss of specific capacitance after 5000 charge–discharge cycles. Therefore, the SC cells fabricated *via* ZnACs (1 : 2) exhibited higher capacitance retention and better long-term stability, and outperformed several reported SC cells with other biomass-derived ACs and electrolytes. Overall, the SCs with ZnACs (1 : 2) carry the potential in filling various loopholes, barring the optimal performance of SCs by providing a cheap, eco-friendly, and highly efficient approach for making SCs for energy storage.

## Summary

In summary, we have prepared ZnACs as electrode materials from cotton shell biowaste and APP films as porous polymer electrolytes prepared using PCL : PVDF-HFP (9 : 1 w/w) and activated with an organic electrolyte (1 M NaClO_4_ in EC : PC). Using the ZnACs (1 : 2) and APP films, we have constructed SC cells and systematically investigated the electrochemical performance of the prepared SC cells. The main results obtained in this work are as follows:

(1) The ZnACs prepared with a mass ratio of 1/2 of cotton shell to ZnCl_2_ showed the largest BET surface area with appropriate porous behaviour, facilitating optimal ion migration making them suitable for SC electrodes.

(2) The activated PP films exhibited a high ionic conductivity of ∼1.5 × 10^−3^ S cm^−1^ at room temperature and a high ESW of ∼7.22 V *versus* Ag.

(3) The operating potential window for the SC cells with the prepared APP film and ZnACs (1 : 2) is 0–2 V.

(4) The SC cells with ZnACs (1 : 2) and an APP film exhibited better electrochemical performance than the SC cells with CmACs and the same APP film.

(v) The SC cells with ZnACs (1 : 2) and an APP film exhibited an excellent long-term cycling stability with only ∼3% loss of specific capacitance.

## Data availability

All possible experimental and analysed results have been included in this manuscript and ESI file.† No other new data have been generated by any further experiments/analyses.

## Author contributions

Saurabh Singh: writing – original draft, methodology, investigation, formal analysis, data curation, conceptualization, final revisions. Yulin Zhang: Raman spectra, long-term stability test, review of the 1st draft. S. A. Hashmi: writing – review & editing, methodology, conceptualization, data analysis. Fuqian Yang: writing – review & editing, methodology, conceptualization, data analysis.

## Conflicts of interest

The authors declare that they have no known competing financial interests or personal relationships that could have appeared to influence the work reported in this paper.

## Supplementary Material

RA-015-D5RA00696A-s001
