# Supplementary material for: Supercapacitors with cotton shell-derived activated carbons and porous polymer electrolyte films
Source: RSC Adv. 2025 Apr 1;15(13):9787–800. doi: 10.1039/d5ra00696a (PMC11959461; doi:10.1039/d5ra00696a)
Supplement: RA-015-D5RA00696A-s001 [file RA-015-D5RA00696A-s001.pdf]

This journal is © The Royal Society of Chemistry 2025

Supplementary Information

1. Photographic image of raw cotton shells

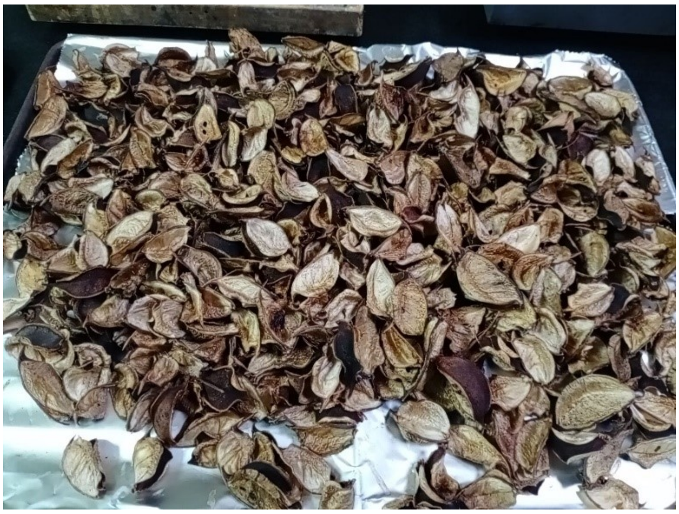

Fig.1: Raw cotton shells after being washed with DI water

2. FESEM images of Porous Polymer(PP) film

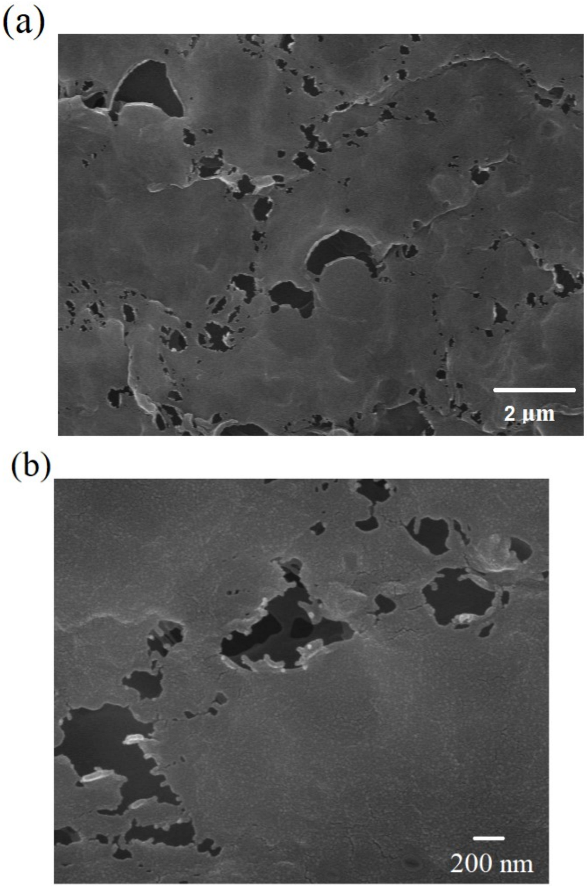

Fig. 2: FESEM images of dried PP film at different magnifications
